# Harnessing Innate Immunity to Treat *Mycobacterium tuberculosis* Infections: Heat-Killed *Caulobacter crescentus* as a Novel Biotherapeutic

**DOI:** 10.3390/cells12040560

**Published:** 2023-02-09

**Authors:** Nancy Gupta, Satish Vedi, Saurabh Garg, Eric Loo, Jie Li, Dennis Y. Kunimoto, Rakesh Kumar, Babita Agrawal

**Affiliations:** 1 Department of Laboratory Medicine and Pathology, Faculty of Medicine and Dentistry, College of Health Sciences, University of Alberta, Edmonton, AB T6G 1C9, Canada; 2 Department of Surgery, Faculty of Medicine and Dentistry, College of Health Sciences, University of Alberta, Edmonton, AB T6G 2S2, Canada; 3 Mitacs, University of British Columbia, Vancouver, BC V6T 1Z3, Canada; 4 Department of Medicine, Faculty of Medicine and Dentistry, College of Health Sciences, University of Alberta, Edmonton, AB T6G 2R7, Canada; 5 ImMed Biotechnologies, Edmonton, AB T6R 2E8, Canada

**Keywords:** tuberculosis, immunotherapy, innate immunity, chemo-immunotherapy

## Abstract

Tuberculosis, caused by *Mycobacterium tuberculosis* (*Mtb*), is a serious and devastating infectious disease worldwide. Approximately a quarter of the world population harbors latent *Mtb* infection without pathological consequences. Exposure of immunocompetent healthy individuals with *Mtb* does not result in active disease in more than 90% individuals, suggesting a defining role of host immunity to prevent and/or clear early infection. However, innate immune stimulation strategies have been relatively underexplored for the treatment of tuberculosis. In this study, we used cell culture and mouse models to examine the role of a heat-killed form of a non-pathogenic microbe, *Caulobacter crescentus* (HKCC), in inducing innate immunity and limiting *Mtb* infection. We also examined the added benefits of a distinct chemo-immunotherapeutic strategy that incorporates concurrent treatments with low doses of a first-line drug isoniazid and HKCC. This therapeutic approach resulted in highly significant reductions in disseminated *Mtb* in the lungs, liver, and spleen of mice compared to either agent alone. Our studies demonstrate the potential of a novel innate immunotherapeutic strategy with or without antimycobacterial drugs in controlling *Mtb* infection in mice and open new avenues for the treatment of tuberculosis in humans.

## 1. Introduction

Tuberculosis (TB) is a global infectious disease caused by *Mycobacterium tuberculosis* (*Mtb*), infecting eight to nine million people and killing two to three million people annually worldwide [1]. Control of TB disease is highly challenging due to four main factors: (1) the ineffectiveness of the current vaccine BCG (Bacillus Calmette–Guérin); (2) poor compliance associated with lengthy treatments with multiple chemotherapeutic drugs that have serious side effects; (3) the prevalence of active TB disease in HIV co-infected individuals with new TB infections, reinfections or latent infections due to immunocompromised status; and (4) the rapid emergence of multi/extensively/totally drug resistant TB (MDR/XDR/TDR-TB). Extensive efforts to discover a new and/or improved TB vaccine have not been successful [1,2,3,4,5,6,7]. Consequently, there is a need to open new avenues and investigate novel immunotherapeutic approaches to combat the expanding TB epidemic and improve treatment outcomes.

One of the potential approaches for treatment and/or cure of chronic mycobacterial infections is antigen-independent immunotherapy, which boosts the host innate immune system to fight disease [8]. The precise contributions and fine-regulation of different innate immune mechanisms needed to fight against TB remain undefined. However, animal and human studies suggest that regulated activation of immune cells promotes mycobacterial containment, while their uncontrolled activation/inflammation causes active disease with severe organ damage [9,10,11,12]. Several host-directed therapies targeting different immune mechanism including cytokines, corticosteroids, vitamin D3, and thalidomide have been explored as potential immunotherapies with limited efficacy [13,14,15,16].

Along these lines, several live or heat-inactivated microbes have shown beneficial effects in preventing the development of, or protection from diseases associated with immunological disorders [17,18,19,20,21,22]. Live and heat-killed lactobacillus species have been evaluated as immunotherapeutics and vaccine adjuvants, however, poor induction of antigen-specific antibody and T cell responses have limited their use [23,24,25,26,27,28,29,30]. In recent years, cumulative evidence has also shown the role of probiotics and commensal bacteria in the induction and/or restoration of regulatory and protective immune responses in the gut [31,32,33]. In addition, some environmental non-tuberculous mycobacterial species have shown efficacy alone or in combination with chemotherapeutics in cancer and *Mtb* infections [34,35,36]. Live *Mycobacterium marinum* and heat-inactivated *Mycobacterium manresensis* have been shown to be effective for immunotherapy in in vitro and in vivo models, respectively. Although exposure to environmental mycobacterial species does not cause infectious disease, they are known to cause various skin lesions, induce severe systemic immune responses, and/or compromise the host’s ability to respond effectively to other pathogens [36,37]. Therefore, novel, safer, host-directed interventions are needed to offer effective immunotherapeutic strategies to induce a clinically relevant immune response against *Mtb*.

In recent years, our understanding of how non-pathogenic bacteria-based immunotherapy stimulates and regulates innate immune mechanisms has opened new avenues to discover novel immunotherapeutic interventions. Many of the microbes tested activate innate immunological sensors, which result in the aberrant production of inflammatory or anti-inflammatory cytokines, leading to either auto-inflammation or recurrent infections [20,21]. As a result, careful selection and identification of such microbial agents are essential. Treatment with potentially pathogenic and non-pathogenic bacteria has been used in both preclinical models and clinically for tumor therapy [21,22,30]. These same strategies may have similar benefits for chronic infections like latent TB.

*Caulobacter crescentus* (*Cc*) is a non-pathogenic freshwater aquatic bacterium that is not known to cause any infections or diseases in mammals [38]. *Caulobacter crescentus* has been comprehensively studied due to its dimorphic life cycle and high expression of an S-layer subunit protein RsaA [38,39,40,41,42,43,44]. Recombinant *Cc* has been reported as a useful expression vehicle due to the presence of an abundant S-layer, which can be genetically modified to express target molecules, antigens, or antibodies [38,39,40,41,42,43,44]. Live *Cc* has been shown to provide antitumor responses in mouse models, however, the basis of this effect was not determined [45].

Both live and inactivated forms of therapeutic bacteria have been explored for TB immunotherapy [34,35,36,37]. However, for an infection like TB, which is strongly associated with immunosuppression, treatment with live bacteria, even with a high safety profile, has the potential to cause a yet unknown infection. Therefore, non-viable microbial agents need to be used for effective immunotherapeutic strategies against TB. Heat-killed *Caulobacter crescentus* (HKCC) has never been previously investigated for its use as an immunotherapeutic intervention. In the present study, we explored the effect of HKCC in controlling *Mtb* using an in vitro THP-1 and an in vivo mouse model of infection, and examined its ability to stimulate local and systemic innate immunity. Our studies demonstrate the tremendous potential of heat-killed *Caulobacter crescentus* (HKCC) as a novel immunotherapeutic agent for the treatment of deadly TB infections, alone and in combination with other chemotherapeutic agents.

## 2. Materials and Methods

### 2.1. Animals

All animal experimental protocols used in this study were approved by the University of Alberta Animal Care and User Committee for Health Sciences (AUP 00000212 and AUP00000279) and conducted in accordance with the guidelines of the Canadian Council of Animal Care. Five to six-week old female BALB/c mice were purchased from Charles River Laboratories (Laval, QC, Canada) and housed in the BSL2/3 animal facility (HSLAS) at the University of Alberta.

### 2.2. Heat-Killed Caulobacter crescentus

*Cc*, containing a p4A723/cmyc plasmid vector with a chloramphenicol resistance marker were grown in an incubator at room temperature (22–27 °C) in PYE medium supplemented with chloramphenicol (2 µg/mL). Logarithmically growing cultures were centrifuged at 6000 rpm for 15 min and the bacterial CFU was determined by measuring OD and confirmed by plating serially-diluted bacterial suspensions on PYE agar. To prepare heat-killed *Caulobacter crescentus* (HKCC), live *Cc* was treated at 80 °C for 60 min, centrifuged, and resuspended in saline at the desired concentrations. To determine viability post heat treatment, the prepared suspension of HKCC was plated on PYE agar plates in serial dilutions. No bacterial colonies grew in these plates, confirming the non-viability of bacterial cells. Every batch of HKCC was tested to confirm non-viability prior to use.

### 2.3. Cytokine Induction in Mice

For in vivo cytokine induction studies, groups of five C57bl/6 male mice were administered with saline or HKCC (50 × 10^6^/mouse) intranasally (30 µL/mouse). Five hours after administration, mice were euthanized and lung washes (1 mL/mouse) were collected and pooled by group.

### 2.4. Dendritic Cell Differentiation from Mouse Bone Marrow

Hind limb bones (tibia and femur) were isolated shortly after the C57bl/6 male mice were euthanized. The bones were flushed with PBS using a 27 G needle and 5 mL syringe. Bone marrow was strained through a 70 µm nylon mesh filter (Fisher Scientific; Ottawa, ON, Canada) and mechanical pressure was applied using the plunger from a 5 mL syringe. Bone marrow cells were washed and red blood cells (RBC) lysed using filtered double distilled H_2_O (ddH_2_O) for 10 s. Cells were then counted and seeded onto a 24-well tissue culture plate at 1 × 10^6^ cells/well in RPMI media supplemented with 5% FBS and 800 U/mL of GM-CSF (Peprotech, Cranbury, NJ, USA). Plates were incubated and the medium was changed every 2–3 days according to the pH indicator of the medium. After 7–9 days of incubation, the loosely attached cells were collected, washed in PBS, and plated at 1 × 10^6^/mL/well in fresh medium with 20 × 10^6^ CFU of HKCC in 24-well tissue culture plates. Supernatant was collected 18–20 h after incubation and frozen for ELISA. Whole cells were stained for CD11c, CD86 (clone GL-1), and CD40 (clone 3/23) with fluorescently labelled antibodies (BioLegend; San Diego, CA, USA). Dendritic cell populations were >80% pure, as determined by CD11c expression.

### 2.5. Enriching for CD49b^+^NK/NKT Cells

C57bl/6 mouse spleens were collected quickly after euthanasia. Single-cell suspensions of splenocytes were then washed with PBS and RBCs lysed with ddH_2_O for 10 s. A magnetic cell separation assay was used to positively select for CD49b expression (clone DX5) with a panNK Cell Isolation Kit (Stemcell Technologies; Vancouver, BC, Canada), before being prepared for plating. NK/NKT cells were obtained from the magnetically labeled fraction and washed before being plated with BMDCs.

### 2.6. BMDC-NK/NKT Cell Co-Culture

NK/NKT cells were enriched with splenocytes isolated from C57bl/6 by mice utilizing the panNK Cell EasySep Kit (StemCell Technologies). The kit separates cells based on the NK cell marker CD49b. The 1 × 10^5^ NK/NKT cells were then co-cultured with 1 × 10^6^ BMDCs in 24-well plates overnight. The supernatant that was collected was frozen until cytokine ELISAs could be performed. In the Transwell plate experiments, a 24-well plate with pore sizes of 0.4 µm was used (Corning; NY, USA). These plates do not allow for cellular contact between the two different cell populations but do allow for the transfer of soluble products.

### 2.7. Cytokine ELISAs

Cytokine ELISAs were performed as per the kit instructions. Mouse ELISA kits for various cytokines were purchased from both eBioscience Inc. (San Diego, CA, USA). Supernatants were diluted with the assay diluent to ensure the level of absorbance would land on the standard curve and plated in triplicate. Absorbance was measured at 450 nm. Absorbance values were compared to an assay-specific standard curve to determine the cytokine concentration. The average cytokine concentrations from triplicate samples were determined along with the standard deviation.

### 2.8. Flow Cytometry Analysis of Surface and Intracellular Markers

A total of 1 × 10^6^ cells from spleen and bronchioalveolar lavage (BAL) from HKCC-treated mice were taken for extracellular staining with multicolor fluorescently labeled mAbs (concentrations according to the manufacturer’s instructions). The cells were incubated with Fc mouse-serum (Sigma-Aldrich, Oakville, ON, Canada) to prevent non-specific binding and washed with fluorescence-activated cell sorter (FACS)-buffer (2% fetal bovine serum in 1 × phosphate-buffered saline (PBS)). After 30 min of incubation with anti-mouse CD3e-FITC, CD4-PECy-5, CD4-APC, CD25-PE-Cy7, CD8a-APC-Cy7, CD69-PECy-5, anti-CD49b-Alexafluor-700, anti-CD11c-FITC, anti-F4/80-Alexafluor-450, anti-CD11b-Alexafluor-700, anti-CD40-PE, anti-CD86-APC, anti-MHC-II-PECy-5, etc. (eBioscience) for extracellular markers at 4 °C, the cells were washed twice with FACS buffer and analyzed using a LSR Fortessa SORP flow cytometer. Among the lymphocyte populations, the CD4^−^CD8^−^ CD3^−^CD49b^+^ and CD4^−^CD8^−^CD3^+^CD49b^+^ lymphocytes were considered as NK cells and NKT cells, respectively. Data analysis was conducted using FACS-DIVA software version 6.2 (Becton Dickinson, Mountain View, CA). Each marker was gated based on its respective isotype-matched control monoclonal antibodies.

### 2.9. In Vitro Human Macrophage Infection of Mycobacteria

THP-1 cells (human monocyte cell line, obtained from ATCC, Rockville, MD, USA) were grown in complete DMEM medium. Cells (0.5 × 10^6^/mL/well) were cultured in medium containing 50 ng/mL of PMA in a 24-well plate for 24 h to allow for differentiation into macrophages. The next day, cells were infected with *M. tuberculosis* (*Mtb*) H37Ra or *M. avium* (0.5 × 10^6^ CFU/well, MOI of 1) for 4 h at 37 °C and then washed with medium to remove the extracellular bacilli. In separate cultures, human peripheral blood mononuclear cells (PBMCs, 4 × 10^6^/mL/well) were incubated in 24-well plates with AIM V medium with or without HKCC (50 × 10^6^ CFU/mL), or LPS (1 µg/mL) for 24 h and the supernatants were collected. THP-1 cells were treated twice every 4 days with the supernatants collected from human PBMCs stimulated with HKCC, LPS, and directly with the control drugs. Five days after the second treatment, cells were lysed and plated on 7H11 agar plates for CFU determination. 

### 2.10. Mycobacterial Challenge in Mice

For the *Mtb* challenge studies, groups of five female BALB/c mice were infected with *Mycobacterium tuberculosis* H37Ra (0.5 × 10^6^ CFU/mouse) intravenously. After 5 days, mice were treated intranasally once weekly for four weeks with HKCC (50 × 10^6^/mouse). Five days after the last treatment (i.e., >5 weeks post infection), mice were euthanized and bacterial loads in the lungs, liver, and spleen were determined by incubating organ homogenates from individual mice on 7H11 Middlebrook agar plates (BD Biosciences). The plates were incubated at 37 °C in ambient air for up to 3–4 weeks prior to counting the colonies. Lung washes, BAL, and spleens were collected to examine the cytokines and activation of immune cells.

In the combined treatment experiment, starting 5 days after infection, mice were treated intranasally with HKCC and sequentially with isoniazid (INH) orally. Three days after the last treatment, mice were euthanized and bacterial loads were determined in the lungs, liver, and spleen.

### 2.11. Statistical Analysis

Data were analyzed by GraphPad Prism software version 8.3.0 (Graphpad Software Inc., Boston, MA, USA). The Student’s *t*-test, and two-way ANOVA were used to determine the significant difference between two groups and multiple groups, respectively. A *p*-value less than 0.05 (*p <* 0.05) with a 95% confidence interval was considered to be statistically significant. The sample sizes for in vitro and in vivo experiments were three and five, respectively. Each *p*-value was adjusted to account for multiple comparisons. The statistical analyses were corrected for multiple comparisons using statistical hypothesis testing using Tukey’s test.

## 3. Results

### 3.1. Intranasal Administration of HKCC Induces Cytokine Production in Mice

**Figure 1:**
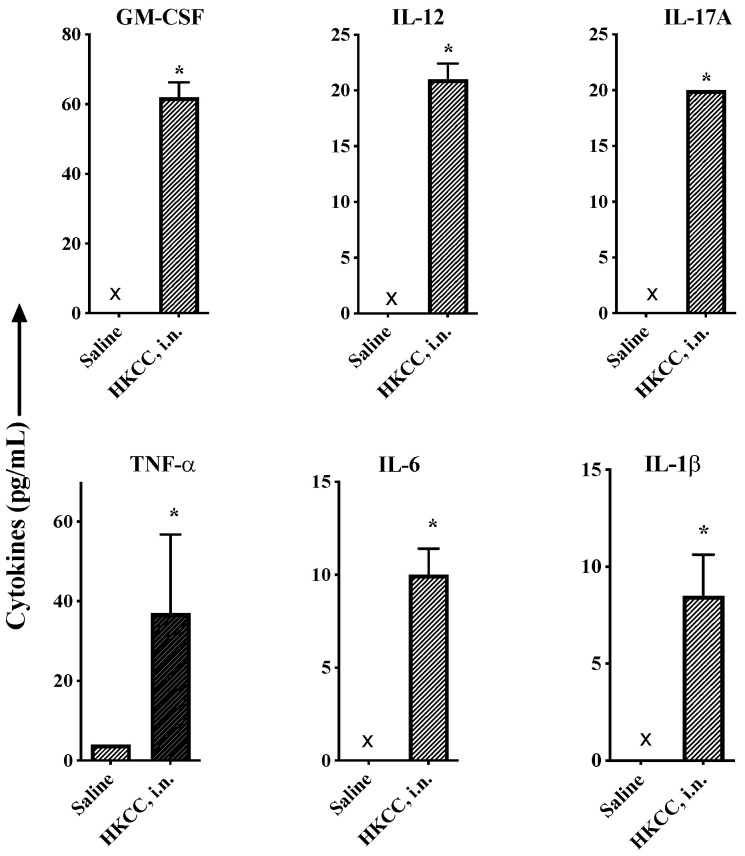
HKCC induces the rapid production of cytokines associated with innate immunity in lungs after intranasal administration in mice. Groups of five C57bl/6 male mice were administered with saline or HKCC (50 × 10^6^ CFU/mouse) intranasally (i.n., 30 µL/mouse). Five hours after administration, mice were euthanized and lung washes (1 mL/mouse) were collected (pooled for a group) and used to determine the presence of cytokines GM-CSF, IL-12, IL-17A, TNF-α, IL-6, and IL-1β using sandwich ELISA. Results are shown as the mean ± SD of triplicate values. * *p* < 0.05, indicates significant difference compared to the corresponding saline control.

In order to investigate whether HKCC can induce cytokine production through immediate innate immunity in vivo, C57bl/6 mice were administered with HKCC (50 × 10^6^ CFU/mouse) intranasally. Five-hours post administration, cytokines were determined in lung washes. HKCC induced the rapid production of GM-CSF, IL-12, IL-17A, TNF-α, IL-6, and IL-1β in mice by the intranasal route (Figure 1). In contrast, saline-treated mice did not show detectable levels of these cytokines (Figure 1), demonstrating a basal level.

### 3.2. HKCC Stimulates Innate Immune Cells and Induces Cross-Talk between NK/NKT Cells and Dendritic Cells

**Figure 2:**
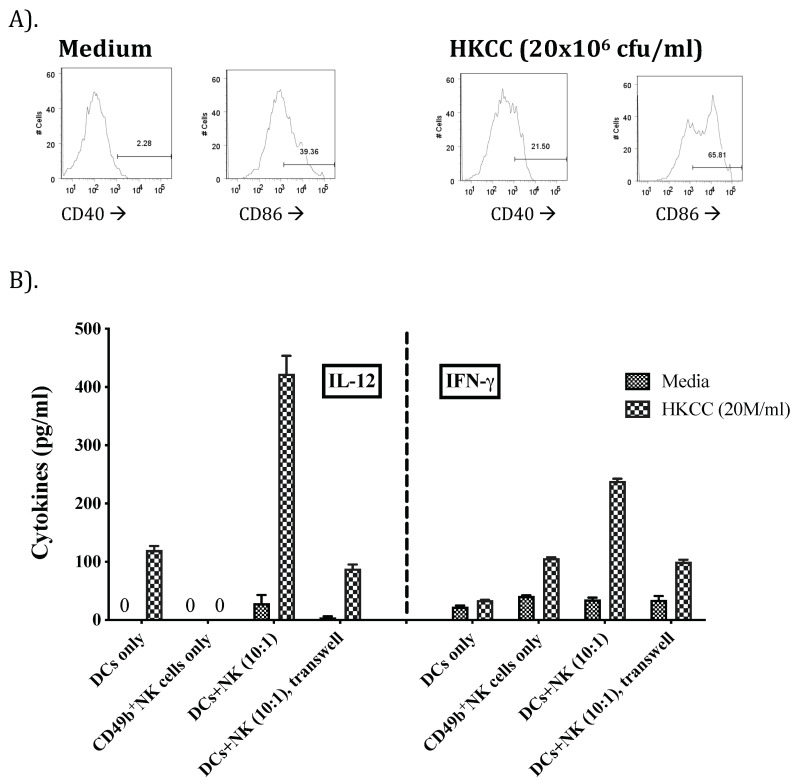
HKCC activates bone marrow derived dendritic cells (DCs) and induces NK-DC cross talk: upregulation of both IL-12 and IFN-γ. (**A**). Bone marrow derived DCs (gated for CD11c expression) were incubated with medium or HKCC (20 × 10^6^ CFU/mL) for 24 h, followed by examining CD40 or CD86 expression by flow cytometry. (**B**). Supernatants were collected from the medium or HKCC (20 × 10^6^ CFU/mL) stimulated co-culture of purified CD49b^+^NK/NKT cells with BMDCs, or contact-separated (Transwell, pore sizes of 0.4 μm) co-culture of BMDCs and NK/NKT cells in triplicate, and were tested for IL-12p70 and IFN-γ production. The data are representative of two separate experiments.

The early/immediate induction of cytokines in the lungs of mice after administration with HKCC suggest a role of innate immune cells in their production. Therefore, we first examined the expression of CD86 and CD40 on bone marrow derived dendritic cells (BMDCs), after incubating them with HKCC, as costimulatory molecules. Interestingly, both of these markers were significantly enhanced compared to the BMDCs stimulated with the medium alone (Figure 2A).

Next, it was important to determine if HKCC stimulates innate cytokine production from DCs and/or NK/NKT cells, and whether HKCC induces cross-talk between DCs and NK/NKT cells. This cross-talk would allow potent innate immune activation in the lungs, with potential to clear *Mtb* in the lungs. Transwell plates with purified CD49b^+^ cells were cocultured with syngeneic BMDCs in compartments either separated by a 0.4 µm filter where only soluble factors were capable of traversing the membrane, or in the same compartment to allow cell-to-cell contact with each other. These plates were incubated with medium alone or HKCC (20 × 10^6^ CFU/mL) for 24 h, followed by examining IL12p70 and IFN-γ in the culture supernatants (Figure 2B). Incubation of BMDCs or purified CD49b^+^NK/NKT cells with HKCC led to a significant induction of IL12p70 or IFN-γ, respectively (Figure 2B). Interestingly, we detected an enhanced IL-12p70 as well as IFN-γ response when the BMDCs were co-cultured with the isolated CD49b^+^NK/NKT cells in the same compartment allowing for cell–cell contact (Figure 2B). However, when the BMDC and NK/NKT cells were separated by a filter insert in the Transwell plate, we did not observe the enhancement IL-12p70 (Figure 2B).

### 3.3. HKCC Inhibits Mycobacterial Growth in Human Macrophages via Host-Mediated Mechanisms

**Figure 3:**
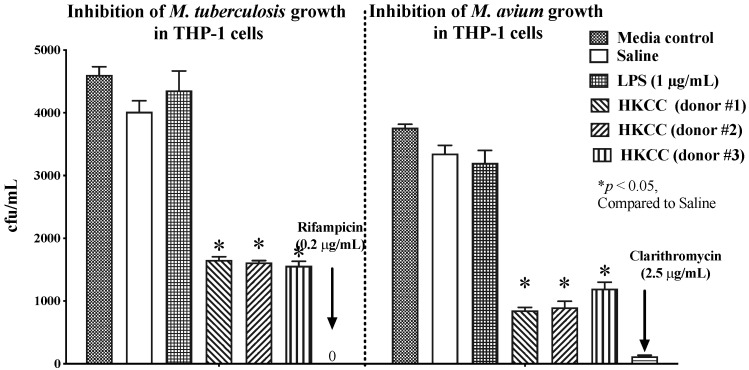
HKCC inhibits intracellular *Mycobacterium tuberculosis* (*Mtb)* and *Mycobacterium avium* (*M. avium)* growth via host-mediated mechanisms in human THP-1 cells. The human monocytic cell line (THP-1 cells) was infected with *M. avium* or *Mtb* H37Ra, followed by two treatments on days 0 and 4 with the supernatants (50%) collected from human PBMCs treated for 24 h with HKCC (50 × 10^6^ CFU/mL), LPS (1 μg/mL), or saline in 24-well plates. As controls, clarithromycin (2.5 µg/mL) and rifampicin (0.2 μg/mL) were added directly to the infected THP-1 cells. Five days after the second treatment, cells were collected, lysed, and plated on 7H11 agar plates to determine the bacterial CFUs. Results are shown from three different donors and represent the mean ± SD of triplicate wells. * *p* < 0.05, indicates significant difference compared to the corresponding saline control.

*Mtb* is an intracellular pathogen, infecting host macrophages, which are the first, phagocytic defense against an invading pathogen. However, infection with *Mtb* incapacitates the ability of macrophages to destroy the bacteria within them [46]. There is ample scientific evidence to support immune mediated clearance of *Mtb* infection [47,48]. Therefore, we sought to determine whether supernatants obtained from human PBMCs stimulated with HKCC would inhibit *Mtb* growth within macrophages. We infected THP-1 macrophages with *Mtb* or *M. avium* and treated the cells with supernatants from PBMC cultures stimulated with HKCC or LPS. Rifampicin and clarithromycin were used as positive controls for *Mtb* and *M. avium*, respectively. Intriguingly, two treatments with supernatants from HKCC-stimulated PBMCs (data from three different donors are shown) led to significant host-mediated inhibition (~60–70%) of mycobacterial load compared to saline-treated supernatants (Figure 3). In contrast, supernatants from PBMCs stimulated with LPS, a TLR4 agonist, did not lead to significant inhibition in intramacrophagic *Mtb* (Figure 3).

### 3.4. Treatment with HKCC Controls Disseminated Mtb Growth in the Mouse Model of Mtb Infection and Is Associated with Both Local and Systemic Innate Immune Stimulation

**Figure 4:**
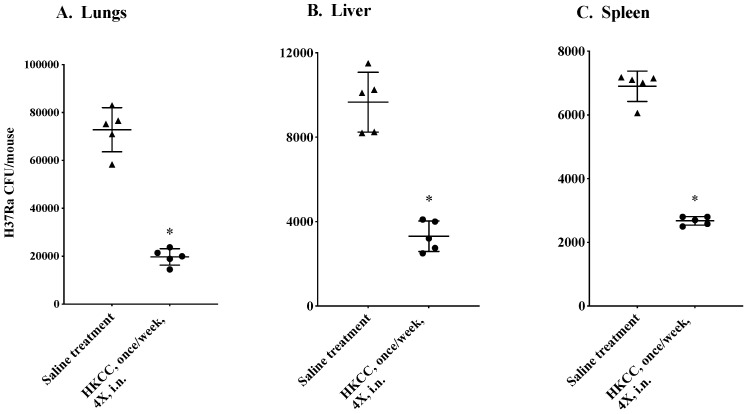
Intranasal administration of HKCC leads to the reduction in disseminated *Mtb* infection in mice. Groups of five BALB/c female mice, infected with *Mtb* H37Ra, were treated with HKCC intranasally (i.n.) once/week for 4 weeks (circles). Control mice were treated with saline (triangles). Five days after the last treatment, mice were euthanized and the lungs, liver, and spleens were collected. Bacterial loads were determined in (**A**) lungs, (**B**) liver, and (**C**) spleen by the CFU assay. All results are shown as the mean ± standard deviation of CFU (colony forming units) from five individual mice. Data are representative of three different repeated experiments. * *p* ≤ 0.05 indicates a significant difference compared to the saline treated mice.

Next, we examined the therapeutic potential of HKCC upon intranasal administration in the *Mtb* (H37Ra) infected mice. Groups of five BALB/c mice infected intravenously with *Mtb* (H37Ra) were treated once weekly intranasally for a total of four times, starting from 3 days post infection. Five days after the last treatment, mice were euthanized and *Mtb* loads were determined in the lungs, liver, and spleen of individual mice by the CFU assay. Remarkably, four intranasal weekly treatments of mice with HKCC substantially reduced the *Mtb* loads in the lungs, liver, and spleen compared to the saline treated group (Figure 4).

**Figure 5:**
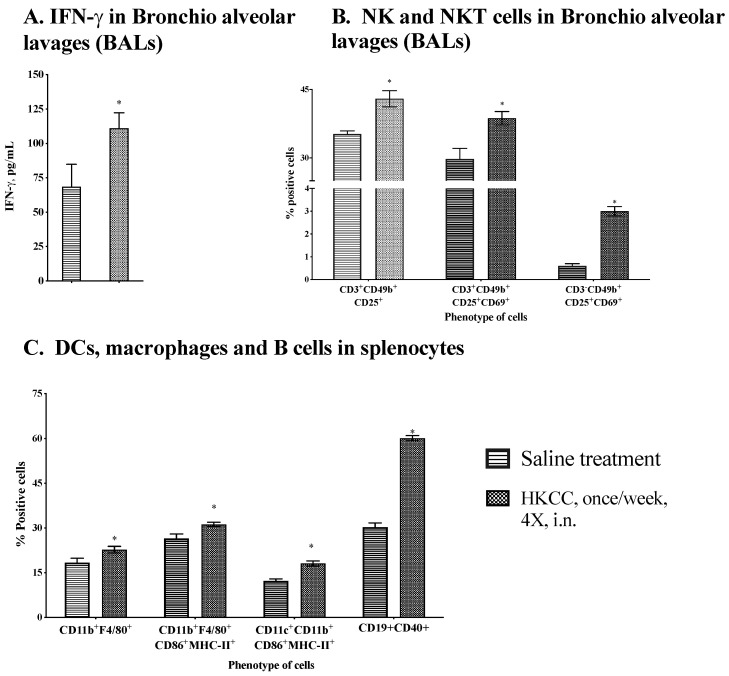

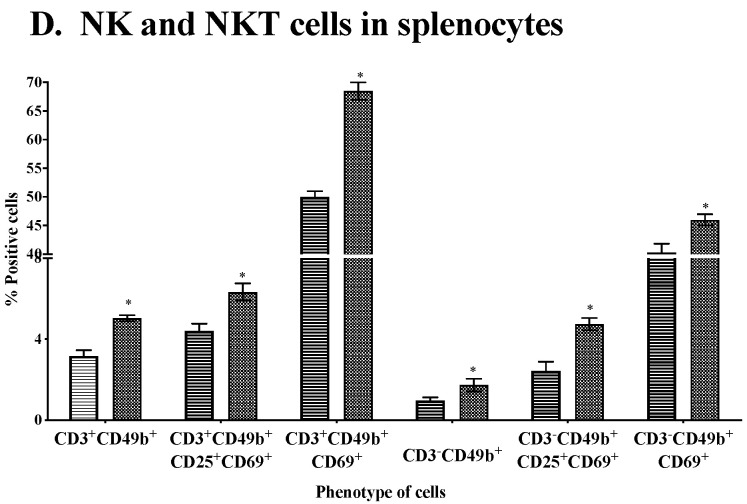
Intranasal administration of HKCC boosts local and systemic immune responses in *Mtb* challenged mice. Groups of five BALB/c female mice infected with *Mtb* H37Ra (0.5 × 10^6^ CFU/mouse) were treated intranasally (i.n.) with HKCC (50 × 10^6^ CFU/mouse) once/week for 4 weeks. Five days after the last treatment, mice were euthanized and bronchio alveolar lavages (BAL) and spleen were collected to determine the immune responses. (**A**) IFN-γ in BAL by ELISA. Mean ± standard deviation of cytokine concentrations from individual mice are shown. Percent positive cells are shown: (**B**) NK and NKT in BAL, (**C**) DCs, macrophages, and B cells in spleen, and (**D**) NK and NKT cells in the spleen. * *p* ≤ 0.05 indicates the significant difference compared to the saline treated mice. Data are representative of three different repeated experiments.

To investigate whether HKCC led to the modulation of local and systemic immune responses in *Mtb* infected mice corresponding to its protective efficacy, we first determined the level of IFN-γ in lung washes. Interestingly, intranasal treatment with HKCC led to significantly (* *p* ≤ 0.05) increased levels of effector cytokine IFN-γ Figure 5A). IFN-γ, an effector molecule known to be produced by adaptive immune effector (T) cells, is also produced in abundance by innate lymphocytes [49]. To determine the effect of HKCC in activating innate immune cells, we first examined the activation of infiltrated NK and NKT cells in BAL by determining CD25 and CD69 expression. Interestingly, coinciding with the presence of IFN-γ in the lung lavages, the percentage of activated NK and NKT cells was significantly higher in mice treated with HKCC by both routes compared to the saline treatment groups (Figure 5B).

Next, we determined the nature of systemic innate immune stimulation upon HKCC treatment in the *Mtb* infected mice. It was interesting to note that HKCC treatment led to upregulated expression of the CD86 and MHC-II molecules on CD11b^+^F4/80^+^ macrophages and CD11b^+^CD11c^+^ DCs, and CD40 expression on B cells (Figure 5C). In addition, treatment with HKCC was associated with increases in the percentage and activation (CD25 and CD69 expression) of NK and NKT cells in the spleen (Figure 5D). Thus, therapy with HKCC in the *Mtb*-infected mice led to significant recruitment and activation of the local and systemic innate immune cells.

### 3.5. Treatment with HKCC in Conjunction with a Low Oral Dose of First-Line Anti-TB Drug Isoniazid (INH) Augments Anti-Mtb Effects in Lungs, Liver and Spleen

**Figure 6:**
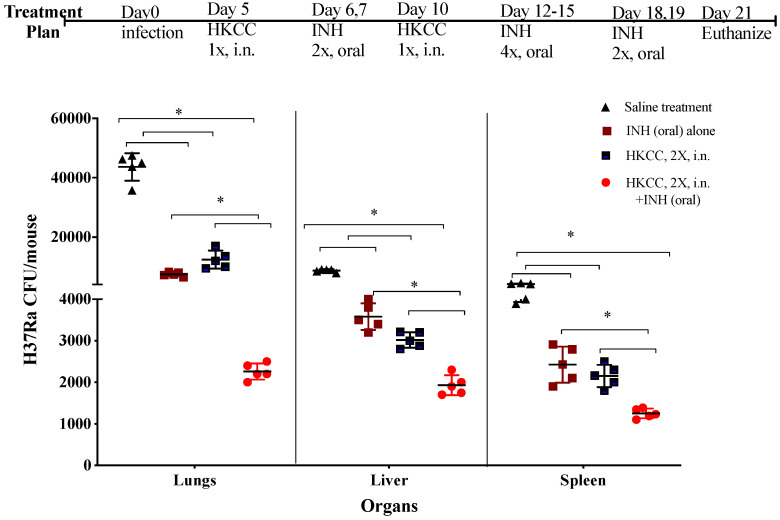
Treatment with HKCC in conjunction with isoniazid (INH) led to a higher reduction in *Mtb* burden in comparison to either agent alone. Groups of five BALB/c female mice, infected with *Mtb* H37Ra (0.5 × 10^6^ CFU/mouse), were treated with HKCC intranasally (50 × 10^6^ CFU/mouse) and isoniazid orally (INH, 1 mg/kg) or PBS using a schedule shown in the figure. Mice were euthanized 2 days after the last treatment. Spleens, lungs, and livers were collected to determine the bacterial loads using the CFU assay. Results are shown as the mean ± standard deviation of CFUs from individual mice. Data are representative of three different repeated experiments. * *p* ≤ 0.05 indicates a significant difference compared to the PBS treated mice.

The potential of immunotherapy of *Mtb* infection could be enhanced if it can be used alongside current anti-TB chemotherapeutics to boost their effectiveness while reducing their dosage and associated side effects. *Mtb*-infected mice were sequentially treated with HKCC (intranasally) and a low oral dose of INH (1 mg/kg) according to the schedule depicted in Figure 6. The concentration of INH was selected based on a previous pharmacokinetic study demonstrating a dose response curve for oral INH [50]. Interestingly, the addition of only two intranasal treatments with HKCC intermittently with a low oral dose of INH led to very promising inhibition (>90%) of the *Mtb* loads in the lungs, liver, and spleen, which was also significantly superior than either agent alone at the same doses and schedules (Figure 6).

## 4. Discussion

TB is an ancient disease, re-emerging worldwide in more dangerous and drug-resistant forms. In the past two decades, significant efforts have been focused on developing an improved vaccine against TB that targets the stimulation of antigen-specific adaptive immune responses. To date, these efforts have not led to measurable success clinically [51,52,53]. Recently, there has been unexpected revelation of the expanding role of innate immune cells in not only providing the immediate first line of defense, but extending beyond that in timelines, memory responses, immune regulation, the control of inflammatory mechanisms and coordinating the downstream adaptive immune response [54,55,56,57,58,59,60]. Our goal is to develop novel immunomodulatory agents of innate immunity that can be used to treat TB as well as a number of other chronic infections. It is prudent now that immunomodulators targeting various innate immune mechanisms are recognized as novel therapeutic agents and not just as adjunct to vaccines. Our studies have uncovered HKCC as just such an example of a promising immunomodulatory/immunotherapeutic agent.

First, we found that intranasal administration of HKCC induces the rapid production of specific cytokines in lung washes measured 5 h after administration, suggesting the potential of HKCC in stimulating innate immunity (Figure 1). Although the exact cellular source of these cytokines was not investigated, the pattern suggests the activation of both myeloid cells such as monocyte/dendritic cells and innate lymphocytes like NK/NKT cells.

Therefore, early induction of multiple cytokines in the lungs led us to examine the activation of DCs by HKCC for the expression of costimulatory molecules CD40 and CD86 (Figure 2A), and the interaction of innate (DCs and NK/NKT) cells (Figure 2B) in vitro. The results demonstrated an important role of HKCC in inducing DCs-NK/NKT cell interaction, resulting in robust synergistic induction of two key cytokines IL-12 and IFN-γ, both of which could potentially orchestrate a successful innate immune response against a pathogen like *Mtb* [61]. NK and NKT cells have recently emerged as playing important roles in immune regulation, immune homeostasis, and memory responses [62,63,64]. Furthermore, classical mechanisms of activation (i.e., absence of MHC1 for NK cells and CD1d-restricted activation of NKT cells) for both NK and NKT cells have recently been shown to be activated by multiple non-classical mechanisms, expanding their role in overall immunity and immune regulation [65]. NK/NKT cells are able to induce the maturation and functional activation of DCs, and NK/NKT-DC cross-talk plays an important regulatory role in both innate and adaptive immunity [66,67]. NK and NKT cells are also known to promote or suppress cell mediated immunity in different conditions [67]. In order to maintain homeostasis, NK and NKT cells can directly induce the maturation of DCs or lysis of immature but not mature DCs [68]. Functionally distinct subsets of NK and NKT cells regulate diverse biological functions [64,65,66,67].

Recently, various innate cytokine pathways have been reported to influence the outcome of *Mtb* infection [63]. The cytokine signature induced by HKCC in mouse lungs upon intranasal administration was indicative of stimulation of heterogeneous innate immune cells (Figure 1). Evidently, supernatants collected from human PBMCs stimulated with HKCC for 24 h showed very promising effects in reducing *Mtb* replication in a human macrophage cell line (THP-1) (Figure 3). The exact role of individual or combinations of cytokines in producing this effect is not clear yet. Interestingly, supernatants collected from PBMCs stimulated with LPS, a TLR4 agonist, did not have the same effect (Figure 3). This observation can be attributed to a broad and distinct range of cytokines/innate cells induced upon stimulation with HKCC compared to the individual TLR4 agonist [69].

Mucosal administration of HKCC in *Mtb*-infected mice led to a remarkable reduction in disseminated infection and bacterial loads in the lungs, liver, and spleen (Figure 4) and was associated with the activation of various innate immune cells including NK and NKT cells (Figure 5). Similarly, although we did not examine the role of the expanding universe of other types of innate lymphoid cells (ILCs) [58,70] in our studies, they cannot be ignored in providing protective immunity against *Mtb* upon treatment with HKCC. NK and NKT are prominent cells of the innate immune response, but their contribution during mycobacterial infections has not been explored much and remains controversial. Depletion of NK cells in mice with antibodies against NK 1.1 and asialo-GM1 enhanced the mycobacterial growth burden in mice [70]. Numbers and functional activity of NKT cells have been shown to be reduced in *Mtb*-infected patients, supporting their role in natural immunity [71,72]. In addition, Kulprannet et al. showed a higher frequency of IL-4 producing NKT cells compared to IFN-γ producing cells in TB patients [73]. Dhiman et al. demonstrated that IL-22 produced from NK cells but not by T cells inhibited the intracellular growth of mycobacteria, and that NK cells have the ability to lyse the Tregs expanded during chronic infection, providing an important role of NK cells in immune defense against *Mtb* [74]. Evidently, our results demonstrate that the in vitro and in vivo effect of HKCC are supported by the activation of NK/ NKT cells and the cytokine production thereof. Taken together, these findings suggest that the immunotherapeutic effects of HKCC in TB are at least partly dependent upon the functional activation of NK and NKT cells. Since innate cells play a critical role in inducing, maintaining, and regulating the downstream adaptive immune responses, the participation of adaptive immune responses in the observed anti-mycobacterial effect of HKCC, although not investigated in the current study, cannot be ruled out.

TB is a resilient chronic infection and is extremely difficult to eradicate from a host [1]. The best strategy for successful treatment and/or cure from TB could be envisioned so that *Mtb* replication can be reduced by chemotherapy, and concurrent immunotherapy can harness the innate immune mechanisms to rid the body of *Mtb* completely. Our experiments using HKCC based immunotherapy in conjunction with a low dose of the first-line anti-TB drug isoniazid (Figure 6) were very promising and set the stage for such treatment regimens. Intriguingly, we observed ~98% reduction in *Mtb* loads in the lungs of mice treated concurrently with HKCC and a low dose of isoniazid compared to 50–70% reduction in the bacterial load with either agent alone. Similarly, in the liver and spleen, a significantly higher reduction in *Mtb* loads was observed upon the concurrent HKCC and isoniazid treatment regimen compared to either agent alone (Figure 6). Collectively, our data demonstrate the tremendous promise of HKCC as a novel immunotherapeutic agent to treat the deadly chronic infectious disease TB, alone and in combination with other anti-mycobacterial drugs.

In conclusion, our results clearly demonstrate the immunotherapeutic potential of HKCC, offering a useful means to stimulate host immunity to protect from and/or to treat wild-type and drug-resistant TB. The induction of efficient host innate immunity by HKCC could possibly be advantageous for the treatment of a wide range of human pathogens.
